# Impact of the Content of Fatty Acids of Oral Fat Tolerance Tests on Postprandial Triglyceridemia: Systematic Review and Meta-Analysis

**DOI:** 10.3390/nu8090580

**Published:** 2016-09-21

**Authors:** Milena Monfort-Pires, Javier Delgado-Lista, Francisco Gomez-Delgado, José Lopez-Miranda, Pablo Perez-Martinez, Sandra Roberta Gouvea Ferreira

**Affiliations:** 1 Department of Nutrition, School of Public Health, University of Sao Paulo, Av. Dr. Arnaldo, 715, São Paulo CEP 01246-904, Brazil; milenamonfort@gmail.com; 2 Lipid and Atherosclerosis Unit, IMIBIC/Reina Sofia University Hospital, University of Cordoba, Av. Menendez Pidal s/n, Córdoba 14004, Spain; delgado-lista@gmail.com (J.D.-L.); jimynaceo@hotmail.com (F.G.-D.); md1lomij@uco.es (J.L.-M.); pablopermar@yahoo.es (P.P.-M.); 3 CIBER Fisiopatologia de la Obesidad y la Nutrición (CIBEROBN), Instituto de Salud Carlos III, Madrid 28029, Spain; 4 Department of Epidemiology, School of Public Health, University of Sao Paulo, Av. Dr. Arnaldo, 715, São Paulo CEP 01246-904, Brazil

**Keywords:** Fat tolerance test, postprandial triglycerides, saturated fatty acids, polyunsaturated fatty acids, monounsaturated fatty acids

## Abstract

Whether the content of saturated (SFA), monounsaturated (MUFA), and polyunsaturated fatty acids (PUFA) could differently influence postprandial triglycerides (TG) is unknown. We examined possible differences in the postprandial TG response to fat tolerance tests (FTTs), in which SFA or unsaturated fatty acids were used. Crossover clinical trials investigating the effects of FTTs containing SFA and unsaturated fats on postprandial triglyceridemia in databases from 1994 until 2016 were searched. Of 356 studies, 338 were excluded and 18 were considered. TG net incremental areas under the curve were calculated using time-points or changes from baseline. Pooled effects of standardized mean differences and I^2^ test were used. Results: In 12 studies, responses to SFA versus PUFA meals, and in 16 studies versus MUFA meals were compared. Over 4 h, no differences between SFA and unsaturated fats were observed. Over 8 h a lower response to PUFA (SMD −2.28; 95% CI −4.16, −0.41) and a trend to lower response to MUFA (SMD −0.89, 95% CI −1.82, 0.04) were detected. FTTs shorter than 8 h may not be sufficient to differentiate postprandial TG after challenges with distinct fatty acids. Clinical significance of different postprandial TG responses on cardiovascular risk in the long-term deserves investigation.

## 1. Introduction

Cardiovascular events are major causes of death worldwide, justifying attempts to improve risk prediction. Evidence suggests that elevated postprandial metabolic changes may be better than fasting conditions to predict cardiovascular risk [1,2]. Demonstration of the role of post challenge glycaemia in morbidity and mortality is facilitated by the standardization of the oral glucose tolerance test [2,3], but that impact associated with lipemic responses is complex since some types of fatty acids can induce postprandial TG at different magnitudes. Despite the evidence of non-fasting triglyceridemia as an independent predictor of atherosclerosis [4,5], how different types of fats consumed modify postprandial triglycerides (TG) concentration remains unclear [6,7].

High-fat meals induce long-lasting TG elevation, limiting the use of fat tolerance tests (FTTs) in clinical investigations [8]. The ideal FTT for the assessment of postprandial TG response should last for 8 to 10 h, but numerous studies have reported data for only 4 h [7,9]. In addition, heterogeneous methodological approaches, in distinct subsets of individuals, have limited comparisons [7,8]. FTTs based on different amounts and types of fatty acids, meal preparations and durations have been proposed [7,8,9]. Therefore, it is still unknown how the composition of fatty meals would affect the cardiovascular risk profile.

Meals containing equal amounts of total fat but with distinct fatty acids composition may have different effects on underlying mechanisms of atherosclerosis such as oxidative stress, inflammation, and lipid disturbances [10,11]. Despite uncertainties regarding the ability of specific dietary fatty acids to influence the TG metabolism [7], it was already described that saturated fatty acids (SFA) intake was able to prolong the raise in plasma TG when compared to unsaturated fatty acids [12,13], and that monounsaturated fatty acids (MUFA) to induce a higher and earlier postprandial responses compared to SFA [13].

In this review and meta-analysis, we investigated whether current evidence supports the observation that SFA-based FTT induces a different postprandial TG response compared to MUFA and/or PUFA.

## 2. Materials and Methods

### 2.1. Types of Studies

All reports that provided measurements of TG concentrations, including mean and standard deviation (SD) or standard error of the mean (SEM) at least at baseline (fasting values), 2 and 4 h during FTT, or changes from baseline were included. Studies that presented only fasting plus one time-value (i.e., fasting and four hours) were not included.

### 2.2. Types of Participants

Since the objective of this meta-analysis was to evaluate the effects of different fatty acids on postprandial TG, regardless of participants’ conditions, we included studies with adult individuals with normal and altered values of plasma TG2.3.

### 2.3. Types of Outcomes

The outcome measure was the standardized mean difference of the net incremental areas under the curve (net iAUC) of TG after FTTs with distinct fatty acids.

### 2.4. Search Methods for Identification of Studies

We searched the electronic databases MEDLINE, Cochrane Database, Web of Science, and EMBASE, from January 1994 until January 2016, for randomized controlled trials and clinical trials, which directly assessed the impact of the acute ingestion of SFA and unsaturated fatty acids (MUFA and/or PUFA) on postprandial TG.

The key words used in the search were: Dietary fat, fatty acids, high-fat meal, olive oil, MUFA, PUFA, saturated fat, omega-3, and *n*-3 fatty acids, plus any of the phrases: Postprandial lipids, postprandial triglycerides/triacylglycerol, postprandial lipemia, and postprandial chylomicron.

### 2.5. Inclusion Criteria

Inclusion criteria were studies with human adults (older than 18 years), published within the period 1994–2016, in English, with sample size larger than six individuals, that used comparisons among different types of fatty acids after FTTs.

Comparisons had to be of SFA with any unsaturated fatty acids using either shakes or mixed meals (carbohydrates and proteins combined with high content of fatty acids). Studies should have fasting and postprandial TG data for at least two time-points. Two and four-hour responses are the most commonly used time-points in FTTs and these were defined for comparisons purposes.

### 2.6. Exclusion Criteria

Reviews and meta-analyses were excluded. Studies that were not investigating unsaturated fat versus SFA (control), simultaneously assessing other dietary or non-dietary interventions, sub-studies of other studies that qualify for inclusion, studies in which the methodology described in the title/abstract showed critical concerns (such as methodological problems), studies in which another active treatment was tested in synergy with fatty acids, studies investigating industrially modified fatty acids, such as hydrogenated unsaturated fatty acids, studies with mixtures of unsaturated fatty acids (MUFA and PUFA in similar amounts), and meals with proportions of saturated to unsaturated fatty acids were not included. We also excluded studies limited to populations with ophthalmologic, obstetric, oncological, gynecologic, renal, neurological, or psychiatric disorders.

### 2.7. Selection of the Studies

**Figure 1:**
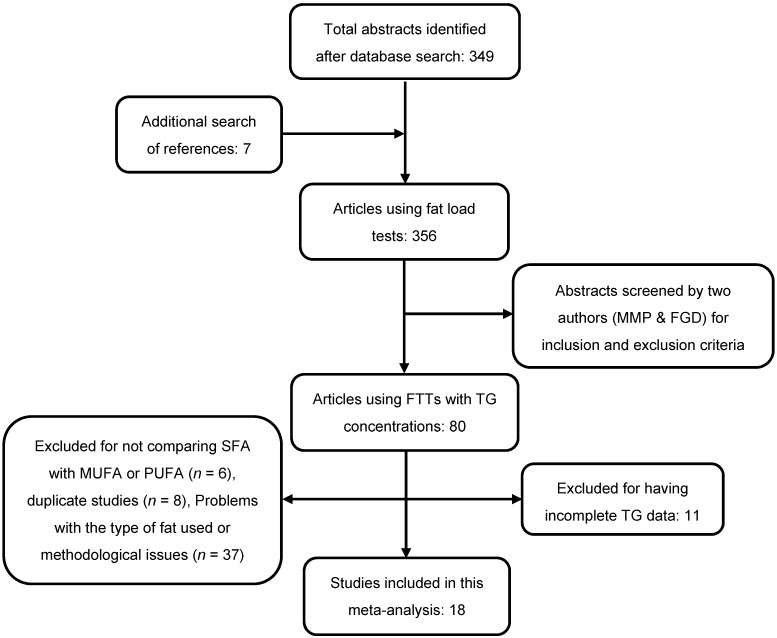
Flowchart of the systematic search strategy and study selection process.

Our search found 356 articles meeting eligibility criteria. From these, 276 were excluded by two authors (MMP and FGD) who independently examined the manuscripts by checking the titles and abstracts, and full-texts, and any problems of disagreement were resolved through group discussion (Figure 1). Full texts of the remaining 80 manuscripts were independently assessed in duplicate to determine inclusion/exclusion based on the quality and risk of bias (see Section 2.8). Sixty-two studies did not meet inclusion criteria (Figure 1). Among those, six studies did not compare SFA with any unsaturated fatty acids, eight were duplicated studies, in 27 mixed fatty acids (PUFA, MUFA and SFA in different proportions) were used and in 10 studies hydrogenated fatty acids were used. Standard deviation or error was not provided in 11 studies.

### 2.8. Assessment of Risk of Bias in Included Studies

Risk of bias was assessed based on the criteria of the Cochrane Collaboration’s Handbook available at the Cochrane website [14]. The criteria considered to determine bias were: appropriate use of a crossover design, randomization of the order of treatment given, bias from carry-over effects, and unbiased data available [14].

### 2.9. Statistical Analysis

Due to differences in the format of the different articles included in this review, a homogenization process was performed, when appropriate: TG concentrations provided in mg/dL were transformed into mmol/L by multiplying by 0.0113. Standard error of the mean (SEM) was transformed to standard deviation (SD) using the formula: SD = SEM × square root of number of sample (*n*).

All TG values were used to calculate the net iAUC according to Wolever [15]. For all studies the standardized mean difference of net iAUC (SMD) was calculated using RevMan 5.3^®^ [16] and the 95% confidence intervals were provided. SMD allows comparing the results from the different measurement values (such as differences from fasting or regular TG values).

Random-effects model was used to estimate the combined effect of the studies, and heterogeneity was assessed using I^2^.

Subgroup analyses were performed to the type of meal, amount of total fat, sex (male participants), and the health status of the study population. Complementary analyses included comparisons of the net iAUC of TG over 2, 6 and over 8 h. Sensitivity analyses were employed for studies outside the funnel plot. All statistical analyses were performed using RevMan^®^ 5.3 [16].

## 3. Results

### 3.1. Final Selection of the Studies, and Review of Their Methodological Details

Table 1 presents the main characteristics of the 18 studies included in this review. We identified 80 potential articles in our search. However, after assessing the inclusion and exclusion criteria, a final selection of 18 studies was taken [10,11,12,13,17,18,19,20,21,22,23,24,25,26,27,28,29,30]. The most frequent cause of exclusion in this final step was the absence of TG values and/or SD or SEM. Of the 18 included, 16 studies compared postprandial TG responses of SFA with MUFA intake and 12 the responses of SFA with PUFA consumption. Ten articles had both comparisons.

This systematic review included 359 participants in the comparison between SFA and MUFA and 223 individuals in the comparison between SFA and PUFA. Sample sizes ranged from 8 to 38 participants and age from 18 to 70 years. Twelve studies included only men [12,13,17,19,20,22,24,25,26,28,29,30] and six included men and women [10,11,18,21,23,27]. A sample of healthy individuals was used in three studies [11,18,21]; in eleven, individuals had no metabolic disorders [10,12,13,17,19,20,22,27,28,29,30]; one with metabolic syndrome [23], one with hypertriglyceridemia [24] and one with hyperlipoproteinemia.

The type of meal used differed among studies. Regarding PUFA comparisons, five studies used shakes [10,13,17,26,27] while for MUFA comparisons, four studies used shakes [10,13,25,26] and 12 used mixed meals [11,18,19,20,21,22,23,24,25,28,29,30]. The amounts of fatty acids also differed among studies. Six studies used less than 60 grams of fat [12,19,22,23,26,27], while another five used 60 grams or more of fat [10,13,17,18,21]. Out of the 18, four studies calculated fat intake based on body weight (1 gram of fat per kilogram of body weight) [20,28,29,30] and two studies calculated the fatty acid intake based on body surface (40–50 g/m^2^ body surface area) [24,25]. When given as food, the main source of fatty acids was butter for the SFA (10 studies) [12,18,19,20,21,28,29,30], olive oil for MUFA (12 studies) [10,18,19,20,21,22,24,25,28,29,30], and two studies used capsules of ω-3 fatty acids in the PUFA meal composition [10,26].

Regarding the protocol duration of the postprandial study, three studies investigated postprandial TG for 4 h [11,13,30], five investigated for 6 h [10,12,21,23,27], one for 7 h [19], seven for 8 h [17,18,22,24,25,26,29], and two for more than 8 h [20,28].

For one study [22], there was no information about randomization but it was considered in the present analysis. The risk of bias of included studies is shown in Table 2.

### 3.2. Clinical Results

#### 3.2.1. SFA *versus* PUFA

**Figure 2:**
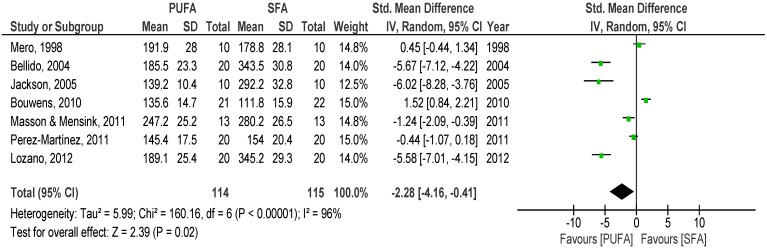
Forest plot of postprandial triglycerides of saturated fatty acids compared to polyunsaturated fatty acids over 8 h.

**Figure 3:**
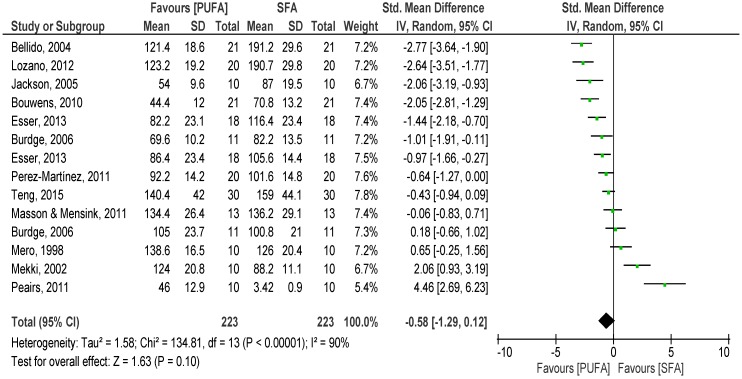
Forest plot of postprandial triglycerides of saturated fatty acids compared to polyunsaturated fatty acids over 4 h.

The overall pooled analysis of the 12 studies comparing the effects of SFA with PUFA on postprandial TG revealed lower SMD of the net iAUC to PUFA meal than to SFA meal over 8 h (*p* = 0.02) (SMD −2.28; 95% CI −4.16, −0.41; I^2^: 96%; Figure 2) and trends of lower response to PUFA meals over 6 h (*p* = 0.10) (SMD −1.04, 95% CI −2.28, 0.20, I^2^: 95%). Over 4 h, a non-significant lower TG response was observed after the PUFA meal (*p* = 0.10) (SMD −0.58, 95% CI −1.29, 0.12, I^2^: 90%, %, Figure 3). Similar findings were observed in the first two hours after meal consumption (*p* = 0.02) (SMD −0.39, 95% CI −0.72, −0.05, I^2^: 66%).

When stratifying by sex, for men the net iAUC over 4 h was lower after PUFA than after SFA meal (*p* < 0.01) (SMD −1.27, 95% CI −1.97, −0.57, I^2^: 87%). Insufficient data for women precluded such analysis In addition, a subgroup analysis of healthy and lean individuals showed lower 4−h iAUC after PUFA than SFA (*p* < 0.01) (SMD −1.48, 95% CI −2.35, −0.61, I^2^: 88%). Furthermore, subgroup analysis for the type of meal over 4 h indicated a trend of lower net iAUC of TG after PUFA compared to SFA when the type of meal used was mixed meals (*p* = 0.07) (SMD −0.94, 95% CI −1.97, 0.09, I^2^: 94%) but not when studies used shakes (*p* = 0.71) (SMD −0.20, 95% CI −1.25, 0.85, I^2^: 90%). When including in subgroup analysis only studies with 8−h data available, the overall pooled effect over 4 h showed a lower TG response to PUFA (*p* < 0.01) (SMD −1.25, 95% CI −1.56, −0.95, I^2^: 90%) compared to SFA meals. Subgroup analysis for the amount of fat was similar to those for the overall pooled sample (data not shown).

#### 3.2.2. SFA *versus* MUFA

**Figure 4:**
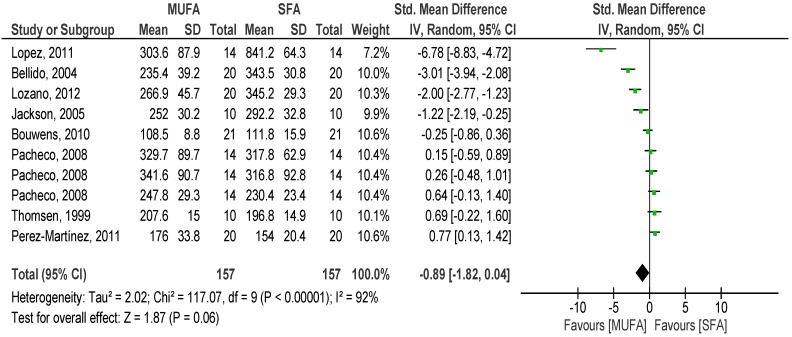
Forest plot of postprandial triglycerides of saturated fatty acids compared to monounsaturated fatty acids over 8 h.

**Figure 5:**
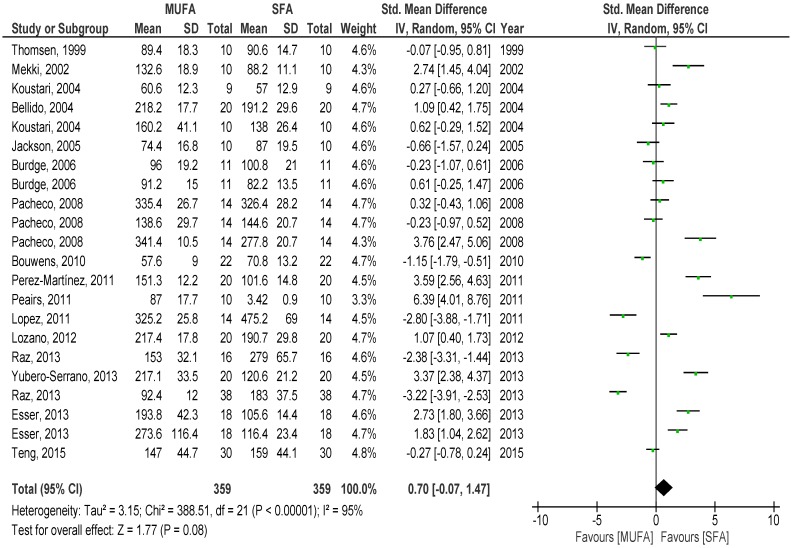
Forest plot of postprandial triglycerides of saturated fatty acids compared to monounsaturated fatty acids over 4 h.

Overall pooled analysis of the TG response to SFA compared with MUFA meal showed that there was a tendency of lower net iAUC of TG to the MUFA over 8 h (*p* = 0.06) (SMD −0.89, 95% CI −1.82, 0.04, I^2^: 92%, Figure 4) compared to SFA meal. Over 4 (*p* = 0.08) (SMD 0.70, 95% CI −0.07, 1.41, I^2^: 95%, Figure 5) and 6 h (*p* = 0.91) (SMD −0.04, 95% CI −0.84, 0.75, I^2^: 93%), no differences between meals were found. Interestingly, over two hours, a non-significant lower iAUC was observed after SFA meal (*p* = 0.27) (SMD 0.22, 95% CI −0.17, 0.61, I^2^: 84%).

In the subgroup analysis for men, no differences in TG responses to SFA and MUFA meals over 4 h (*p* = 0.25) (SMD 0.52, 95% CI −0.36, 1.41, I^2^: 94%) were found. Insufficient data precluded specific analysis for women. When stratifying for healthy lean individuals no differences between the two dietary fats over 4 h (*p* = 0.81) (SMD −0.13, 95% CI −0.92, 1.17, I^2^: 95%) were observed. Considering the meal type subgroups, overall pooled effects of studies using shakes indicated a tendency of lower postprandial TG response to SFA meal over 4 h (*p* = 0.06) (SMD 1.46, 95% CI −0.05, 2.96, I^2^: 95%), while the pooled effect of those using mixed meals showed no difference between fatty acids contents (*p* = 0.58) (SMD 0.28, 95% CI −0.66, 1.22, I^2^: 94%). Subgroup analysis including only studies with 8-h data available showed no difference between meals over 4 h (*p* = 0.36) (SMD 0.46, 95% CI −0.53, 1.46, I^2^: 93%) and subgroup analyses for the amount of fat (<60 grams and >60 grams of total fat) showed no differences between the two type of fats (data not shown).

Two sensitivity analyses were performed to investigate whether the inclusion of individuals with dyslipidemia affected the results. After excluding study by Pacheco et al. [24] from the analysis, no differences were observed in the results over 4 and 6 h. However, over 8 h, the net iAUC after MUFA meals was significantly lower compared to SFA without this study (*p* = 0.03). When the exclusion effect of another study (Lopez et al. [25]) was analyzed no differences were observed over 4, 6 and 8 h. Exclusion of both studies resulted in a non−significant lower TG response after MUFA over 8 h (*p* = 0.17) (SMD −0.82, 95% CI −1.98, 0.34, I^2^: 92%).

Forest plots of postprandial TG of SFA versus PUFA and versus MUFA over 6 h are available as supplemental material (available online at www.mdpi.com/2072-6643/8/9/580/s1), as well as the funnel plots of the studies included in the SFA versus PUFA and SFA versus MUFA comparisons.

## 4. Discussion

This review and meta-analysis aimed at investigating and quantifying the effects of FTT using meals enriched with SFA, PUFA, and/or MUFA on postprandial TG. Our hypothesis that fat challenges containing SFA, MUFA, or PUFA could induce a response of different magnitudes in TG concentrations over 4 h was not supported by the present study. However, overall polled effects showed lower TG responses to unsaturated fatty acids over 8 h when compared to SFA; this indicates that FTTs shorter than 8 h may lack the necessary sensitivity to discriminate differences in postprandial TG after challenges with distinct fatty acids.

Despite the recognition that fat loads provoke a raise in TG concentrations for hours, and that hypertriglyceridemia is an independent cardiovascular risk factor [1,4,5,7], how dietary fatty acids could differently increase its concentration affecting the cardiovascular risk requires investigation. Our study added that SFA and unsaturated fatty acids have different acute impacts in postprandial triglyceridemia over 8 h, which could imply in a worse prognostic for cardiovascular outcomes. These findings are in agreement with cohort studies in which non-fasting TG was associated with fatal and non-fatal cardiovascular events in men and women [4,5,31]. In fact, the deleterious role of chronic high consumption of SFA, mainly lauric, palmitic, and mystiric acids, in lipid and glucose metabolism, has been consistently demonstrated in animal models and in humans [32,33,34,35]. SFA acts as ligand of toll-like receptor 4 [TLR-4] that plays a crucial role in the innate immune system, since it triggers inflammatory pathways by augmenting the expression of TNF-α, IL-6, and MCP-1 genes and deteriorating insulin signaling [36,37,38]. In contrast, chronic intake of unsaturated fatty acids has been associated with cardioprotective effects [39,40]. MUFA intake has been shown to increase HDL-cholesterol and reduce inflammatory markers [39], while ω-3 PUFA has been linked with anti-inflammatory effects [40]. Our observation of a higher 8-h postprandial response to SFA-enriched meals suggests that such long-term stimuli could induce a state of low-grade inflammation and insulin resistance. As cytokine-induced insulin resistance in the liver deteriorates lipid and glucose homeostasis, this might contribute to increase cardiovascular in the long-term.

Our current analyses of the effects of the long-duration FTTs could help understanding how deleterious could be repetitive meals rich in SFA to the cardiovascular risk profile. Since humans spent most of the day in fed state, a cumulative effect of SFA-enriched diet on postprandial TG on a daily basis could lead to a sustained pro-inflammatory state and insulin resistance and atherogenesis in the long-term.

The variety in the duration of postprandial tests may have influenced the results of this systematic review and meta-analysis. FTTs with 4-h duration have been a common procedure in clinical trials due to participants’ compliance and financial and methodological concerns [1,7,8,9,41]. Our overall pooled analysis effect suggests that assessing TG for 4 h is not enough to observe differences between meals with distinct fatty acids. For the SFA versus PUFA comparison, overall pooled effects for 4 h indicated a non-significant lower response after the PUFA meal. Over 6 h we also observed a tendency of lower TG net iAUC after the unsaturated fat compared to the SFA, and a significantly lower response over 8 h. Even considering the high heterogeneity among the studies, our meta-analysis indicated that PUFA meal results in less pronounced TG postprandial response over 8 h.

When SFA and MUFA meals were compared, we observed a non-significantly lower net iAUC after SFA over 4 h, while over 6 h both meals had similar impact on postprandial TG. However, examining for a longer period, over 8 h, a tendency (*p* = 0.06) of lower TG response to MUFA meals was observed. This may be interpreted as an earlier and higher peak of TG after MUFA than SFA in the first hours of postprandial test [41,42]. Actually, a non-significant greater iAUC after MUFA compared to SFA over two hours was observed. Endorsing this, unpublished data of our group showed different patterns of the postprandial lipemia, with a smaller SFA peak than MUFA at 2 h, but remaining higher up to 8 h. Considering the evidence that maintenance of prolonged elevation in plasma TG may favor atherogenesis [40,41], SFA-enriched meals should be restricted at least for individuals at cardiovascular risk. Whether a shorter exposure of the endothelium to an accentuated hypertriglyceridemia—such as that provoked by MUFA consumption—would have a less harmful effect deserves investigation [7,8,41,42]. In addition, lower TG response to PUFA than to SFA during the 8-h period was detected in our meta-analysis, which is in agreement to our unpublished data (Figure S5; available online at www.mdpi.com/2072-6643/8/9/580/s1).

Supposing that sex could influence postprandial lipemia [7,41,42], we performed a subgroup analysis that showed that in men TG response to PUFA consumption was significantly lower than to SFA meals over 4 h. These results suggest that differences in populations’ characteristics of the studies included in this review may have affected the statistical significance of the overall pooled analysis. Most studies were conducted in men probably in order to minimize estrogen-related confounders [41,42,43]; therefore, the lack of data in women did not allow subgroup analysis for this sex.

Similar results were also seen when stratifying by the absence of any non-communicable diseases. Subgroup analysis involving normal weight, healthy individuals showed lower TG response only to PUFA compared to SFA over 4 h. This finding is in accordance with the previous evidence that obese individuals respond differently to high-fat meals [7,8,10] and that diseases—such as diabetes mellitus and dyslipidemia—influence the degree of postprandial lipemia [8,40,42].

Stratification according to the meal preparation suggests an influence in the overall pooled effects. SFA-enriched shakes tended to promote a less pronounced TG response than MUFA over 4 h. However, when mixed meals were used, no difference between fatty acids ingested was seen. This finding could be partially explained by differences in the absorption rate of fatty acids (liquid *versus* solid) and metabolism, as well as to the ingestion of other nutrients such as proteins and carbohydrates that may have affected the postprandial TG response [40,41]. In fact, there is previous evidence that mixed meals can delay nutrients’ absorption and interfere in postprandial lipemia [11,18,19,20,21,22,24,25,28,29,30,41,42]. We call attention that more details on the subtypes of PUFA and MUFA consumed would be desirable, since some PUFA, like the ω-6, was associated with pro-inflammatory [44] while the ω-3 with cardioprotective effects [38,40,44].

We also performed a sub-group analysis in which we just compared the 4 and 8-h data when both values were available in the studies. The results were similar to those found for the overall pooled analysis, indicating that the lower TG response over 8 h to the unsaturated fatty acids could not be attributed to the availability of articles including 8-h data.

Sensitivity analysis for the MUFA versus SFA comparisons indicated that two studies had strong influence in the results [24,25]. The exclusion of the latter study reduced the borderline significance, while excluding the former in which 28 hypertriglyceridemic and 14 healthy individuals were included, the significance of the 8-h response changed, favoring MUFA meals.

To our knowledge, this is the first systematic review and meta-analysis showing that distinct composition of fatty acids offered in FTTs has different impacts on postprandial triglyceridemia. Not only the postprandial lipemia per se but also its impact on inflammatory status might play an important role in the atherogenesis. Whether and how much the shape of the curve and the magnitude and duration of the postprandial TG would affect inflammation is still unclear. Answers to these questions should help in the debate regarding the impact of hypertriglyceridemia as a diagnostic and prognostic tool in the cardiovascular risk assessment.

Our study has a limitation related to the use of plot information for some TG values. Only few authors returned our contact asking for TG values and their SD. We did not have access to some information such as hourly concentrations and time to peak, impeding to speculate on the clearance of fatty acids. On the other hand, a major strength of our meta-analysis is the relatively high number of studies included and the use of net incremental AUC, which allows comparisons among studies with different protocols. The heterogeneity level observed in our meta-analysis was already reported in others using TG responses to meals [9,45]. Despite our attempts to reduce heterogeneity, this unexplained high rate remained in our analyses. This may be explained by the variability of TG, different procedures in test meals and by study population characteristics. Most studies were conducted in men and in European countries [12,13,17,18,19,20,21,22,24,25,26,27,28,29,30], what did not prevent high heterogeneity. Due to complex experimental design, FTTs are normally performed in a small group of individuals, limiting the overall sample size analyzed.

## 5. Conclusions

In summary, our systematic review and meta-analysis support that FTTs including SFA, MUFA and PUFA over 4 h do not have different impacts on postprandial TG concentrations. However, tests lasting for 8 h are able to differentiate responses to saturated and unsaturated fatty acids meals, although the long-term significance of different postprandial TG responses on cardiovascular risk is unknown. We conclude that, in order to differentiate postprandial TG after challenges with distinct fatty acids, FTTs should be as long as 8 h. Studies with similar methodology are necessary to clarify whether saturation level of fatty acids and/or other properties could influence postprandial TG responses. In parallel, consequences of sustained hypertriglyceridemia are still under investigation.

## Figures and Tables

**Table 1:** Main characteristics of included studies in the review.

Study, Year, Country	Study Sample	Meal Type	Energy Content in the Test	Amount of Fatty Acids	Amount and Type of SFA	Amount and Type of MUFA	Amount and Type of PUFA	% Fat Meal Test	Times of TG Data	Type of TG Measure
Mero et al., 1998, Finland	10 men; Mean age: 36.8 ± 3.1 years; Non-obese; Without health problems	Liquid meal	665/616 kcal	63 g	63 g whipping cream	-	63 g soybean oil	85%	0, 3, 4, 6 and 8 h	Plasma TG (mmol/L)
Thomsen et al., 1999, Denmark	5 men + 5 women; Mean age: 23 ± 2 years; No information about BMI; Healthy	Meal	No info	80 g	100 g butter	80 g olive oil	-	No info	0, 1, 2, 3, 4, 5, 6, 7 and 8 h	Plasma TG (mmol/L)
Mekki et al., 2002, France	10 men; Age range: 20–29 years; Mean BMI 22.1 kg/m^2^; Without health problems	Meal	No info	40 g	40 g butter	40 g olive oil	40 g sunflower	No info	0, 1, 2, 3, 4, 5, 6 and 7 h	Changes from baseline in (mmol/L)
Bellido, 2004, Spain	20 young male adults; No age or BMI information; Without health problems	Meal	50%–66% of the subjects’; daily normal intake of calories	1 g/kg of body weight (65%)	38% SFA (butter)	38% MUFA (olive oil)	16% PUFA (walnut)	65%	0, 1, 2, 4, 6, 8.5 and 11 h	Plasma TG (mg/dL)
Koustari et al., 2004, Greece	10 men + 10 women; Age range: 18–40 years; BMI <27 kg/m^2^; Healthy	Meal	1100 kcal	60 g (48%)	32 g butter	40 g (olive oil)	-	48%	0, 1, 2, 3, 4, 5 and 6 h	Plasma TG (mmol/L)
Jackson et al., 2005, UK	10 men; Mean age: 48 ± 9 years; Mean BMI: 25 ± 3 kg/m^2^; Without health problems	Meal	1000 kcal	50 g	50 g (palm + cocoa)	50 g (olive oil)	50 g safflower oil	No info	0, 1, 2, 3, 4, 5, 6, 7 and 8 h	Plasma TG (mmol/L)
Pacheco et al., 2008, Spain	42 men; Age range: 21-38 years; BMI <27 kg/m^2^; 28 hypertriglyceridemic (14 normotensives and 14 hypertensives) and 14 healthy men	Meal	885 kcal	40 g/m^2^ body surface area	76.8 to 79.2 g of high palmitic sunflower oil	76.8 to 79.2 g of refined olive oil	-	72% fat	0, 2, 4, 6 and 8 h	Changes from baseline (mmol/L)
Lopez et al., 2011, Spain	14 men; Mean age: 33 ± 7 years; Mean BMI: 24.2 ± 5.1 kg/m^2^; With hyperlipoproteinemia	Meal	800 kcal	50 g/m^2^ body surface area	65.3% butter	81% refined olive oil	-	72%	0, 1, 2, 3, 4, 5, 6, 7 and 8 h	Plasma TG (mmol/L)
Masson and Mensinsk, 2011, The Netherlands	13 men; Age range: 18–70 years; BMI: 25–30 kg/m^2^; Without health problems	Meal	978/1015 kcal	50 g	50 g butter	-	40 g margarine + 10 g safflower oil	51%	0, 2, 4 and 6 h	Changes from baseline in (mmol/L)
Bouwens et al., 2010, The Netherlands	21 men; Age range: 19–27 years; BMI range: 18–27 kg/m^2^	Shake	2824 kJ/675 kcal	55 g	70% SFA (butter)	80% MUFA (High oleic sunflower oil)	65% PUFA (40% DHA)	73%	0, 2, 4, 6 and 8 h	Changes from baseline in (mmol/L) (author provided data)
Burdge et al., 2010, UK	11 women + 11 men; Age range: 50–65 years; BMI: 20–30 kg/m^2^; Without dyslipidemia	Shake	No info	47–55 g	38% SFA	43% MUFA	25% PUFA	No info	0.5, 1, 1.5, 2, 2.5, 3, 4, 5 and 6 h	Changes from baseline (mmol/L)
Peairs et al., 2011, USA	11 adults; Mean age: 31.3 ± 3.3 years; BMI >27 kg/m^2^; Without health problems	Shake	1267 kcal	85 g	59% refined palm oil	59% refined olive oil	59% refined olive oil + 4 g omega-3	59%	0, 1, 2, 4 and 6 h	Plasma TG (mmol/L)
Perez-Martínez et al., 2011, Spain	20 men; Mean age: 22 years; BMI: 24.5 kg/m^2^; Without health problems	Meal	60% daily intake	1 g/kg of body weight	35% SFA (butter)	36% MUFA (olive oil)	16% PUFA (walnuts)	60%	0, 1, 2, 3, 4, 5, 6, 8.5 and 11 h	Plasma TG (mmol/L)
Lozano et al., 2012, Spain	21 men; Mean age: 23 years; BMI <30 kg/m^2^; Without health problems	Meal	No info	1 g fat/kg body weight	60% fat (35% SFA—butter)	60% fat (38% MUFA—extra-virgin olive oil)	60% fat (16% PUFA—walnuts)	60%	0, 1, 2, 3, 4, 5, 6, 7 and 8 h	Plasma TG (mmol/L)
Esser et al., 2013, The Netherlands	36 men; Age range: 50–70 years; 18 lean + 18 obese; Without health problems	Shake	990 kcal	95 g	54% total fat (palm oil)	83% total fat (high oleic sunflower oil)	40% total fat (n 3) (40 g palm oil + 55 g marinol)	88%	0, 2 and 4 h	Changes from baseline (mmol/L)
Raz et al., 2013, Israel	16 men + 38 women; BMI <25.9 kg/m^2^; Healthy	Meal	1161 kcal	74 g	56% (24 g SFA)	56% (51 g MUFA)	-	56%	0, 2 and 4 h	Plasma TG (mg/dL)
Yubero-Serrano et al., 2013	10 men + 10 women; age ≥65 years; BMI: 20–40 kg/m^2^; Without health problems	Meal	No info	0.7 g fat/kg of body weight	22% SFA (butter)	24% MUFA (olive oil)	-	38%	0, 2 and 4 h	Plasma TG (mg/dL)
Teng et al., 2015, Malasya	15 men + 15 women; Mean age:33.8 ± 1.7 years; Mean BMI: 30.9 ± 0.8 kg/m^2^; With metabolic syndrome but without chronic diseases	Meal	855 kcal	50.9 g	Palm olein 22.9 g SFA	High-oleic sunflower oil 42.5 g MUFA	Sunflower oil 25.7 g PUFA	-	0, 30 min, 1, 2, 3, 4, 5 and 6 h	Plasma TG (mmol/L)

**Table 2:** Risk of bias of included studies in the review.

Risk of Bias for Cross-over Clinical Trials				
Study	Was use of a crossover design appropriate?	Is it clear that the order of receiving treatments was randomized?	Can it be assumed that the trial was not biased from carry-over effects?	Are unbiased data available?
Mero et al., 1998, Finland	Yes	Yes	Yes	No
Thomsen et al., 1999, Denmark	Yes	Yes	Yes	Yes
Mekki et al., 2002, France	Yes	Yes	Yes	Yes
Bellido et al., 2004, Spain	Yes	Yes	Yes	Yes
Koustari et al., 2004, Greece	Yes	Yes	Yes	Yes
Jackson et al., 2005, UK	Yes	No	Yes	Yes
Pacheco et al., 2008, Spain	Yes	Yes	Yes	Yes
Lopez et al., 2011, Spain	Yes	Yes	Yes	Yes
Masson and Mensinsk, 2011, The Netherlands	Yes	Yes	No	Yes
Bouwens et al., 2010, The Netherlands	Yes	Yes	Yes	Yes
Burdge et al., 2010, UK	Yes	Yes	Yes	Yes
Peairs et al., 2011, USA	Yes	Yes	Yes	No
Perez-Martínez et al., 2011, Spain	Yes	Yes	Yes	Yes
Tholstrup et al., 2011, Denmark	Yes	Yes	Yes	Yes
Lozano et al., 2012, Spain	Yes	Yes	Yes	Yes
Esser et al., 2013, The Netherlands	Yes	Yes	Yes	Yes
Raz et al., 2013, Israel	Yes	Yes	Yes	No
Yubero-Serrano et al., 2013, Spain	Yes	Yes	Yes	Yes
Teng et al., 2015, Malaysia	Yes	Yes	Yes	Yes

## Supplementary Materials

The supplementary materials are available online at http://www.mdpi.com/2072-6643/8/9/580/s1, Figure S1: Funnel plot of postprandial triglycerides of saturated fatty acids compared to polyunsaturated fatty acids over 4 h, Figure S2: Funnel plot of postprandial triglycerides of saturated fatty acids compared to monounsaturated fatty acids over 4 h, Figure S3: Forest plot of postprandial triglycerides of saturated fatty acids compared to polyunsaturated fatty acids after 6 h, Figure S4: Forest plot of postprandial triglycerides of saturated fatty acids compared to monounsaturated fatty acids after 6 h, Figure S5: Triglycerides response after oral fat test with meals rich in SFA, PUFA and MUFA (data presented as mean ± 95% confidence intervals) (unpublished data). Data from 40 individuals subjected to three fat tolerance tests with saturated (SFA), monounsaturated (MUFA) and polyunsaturated (PUFA) fatty acids.
