# Supplementary material for: Impact of the Content of Fatty Acids of Oral Fat Tolerance Tests on Postprandial Triglyceridemia: Systematic Review and Meta-Analysis
Source: Nutrients. 2016 Sep 21;8(9):580. doi: 10.3390/nu8090580 (PMC5037564; doi:10.3390/nu8090580)
Supplement: Supplementary file 1 [file nutrients-08-00580-s001.docx]

Supplemental Materials: Impact of the Content of Fatty Acids of Oral Fat Tolerance Tests on Postprandial Triglyceridemia: Systematic Review
and Meta-Analysis

Milena Monfort-Pires, Javier Delgado-Lista, Francisco Gomez-Delgado, José Lopez-Miranda, Pablo Perez-Martinez and Sandra Roberta Gouvea Ferreira


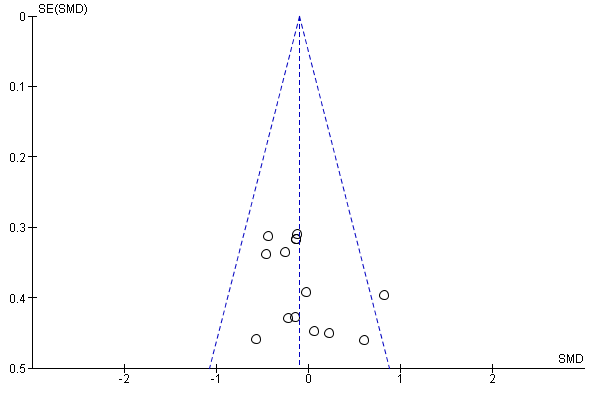


**Figure S1.** Funnel plot of postprandial triglycerides of saturated fatty acids compared to polyunsaturated fatty acids over 4 h.


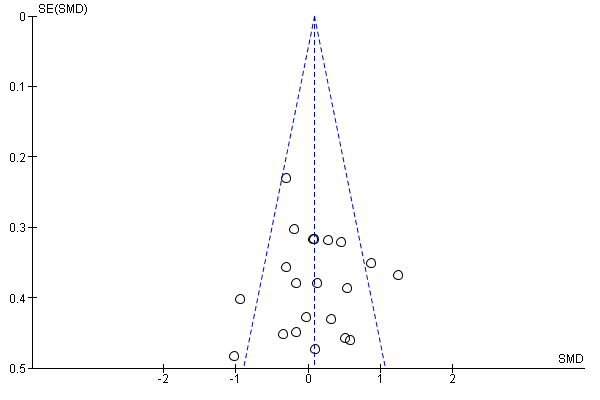


**Figure S2.** Funnel plot of postprandial triglycerides of saturated fatty acids compared to monounsaturated fatty acids over 4 h.

**Figure S3.** Forest plot of postprandial triglycerides of saturated fatty acids compared to polyunsaturated fatty acids after 6 h.

**Figure S4.** Forest plot of postprandial triglycerides of saturated fatty acids compared to monounsaturated fatty acids after 6 h.


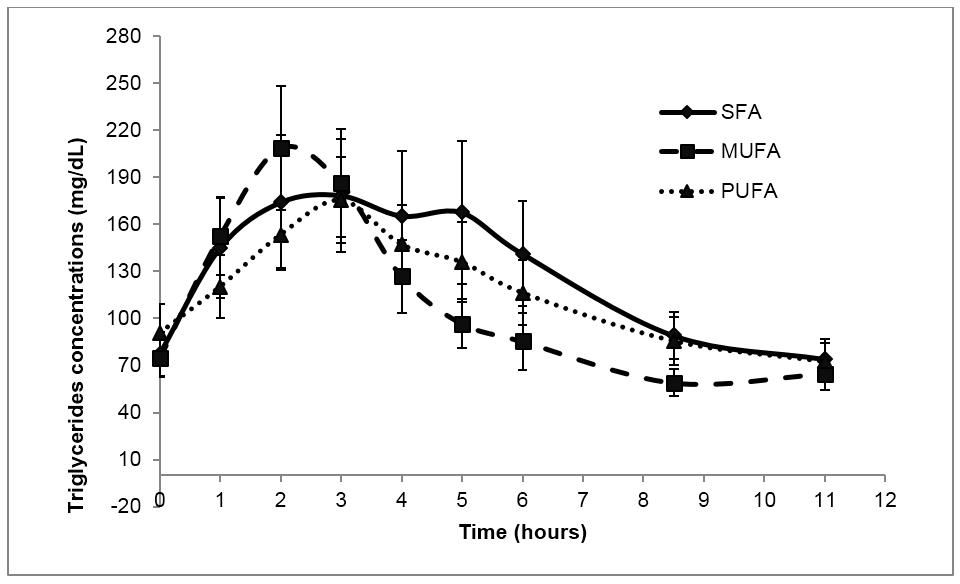


**Figure S5.** Triglycerides response after oral fat test with meals rich in SFA, PUFA and MUFA (data presented as mean ± 95% confidence intervals) (unpublished data). Data from 40 individuals subjected to three fat tolerance tests with saturated (SFA), monounsaturated (MUFA) and polyunsaturated (PUFA) fatty acids.
